# Association between *IRS1* Gene Polymorphism and Autism Spectrum Disorder: A Pilot Case-Control Study in Korean Males

**DOI:** 10.3390/ijms17081227

**Published:** 2016-07-29

**Authors:** Hae Jeong Park, Su Kang Kim, Won Sub Kang, Jin Kyung Park, Young Jong Kim, Min Nam, Jong Woo Kim, Joo-Ho Chung

**Affiliations:** 1 Kohwang Medical Research Institute, School of Medicine, Kyung Hee University, Seoul 02447, Korea; hjpark17@gmail.com (H.J.P.); skkim7@khu.ac.kr (S.K.K.); 2 Department of Neuropsychiatry, School of Medicine, Kyung Hee University, Seoul 02447, Korea; menuhin@hanmail.net (W.S.K.); parkdawit@naver.com (J.K.P.); jimmypage@nate.com (Y.J.K.); psyjong@gmail.com (J.W.K.); 3 Seoul Metropolitan Eunpyeong Hospital, Seoul 06801, Korea; passion17@hanmail.net

**Keywords:** autism spectrum disorder, insulin receptor substrate, single nucleotide polymorphism, insulin-like growth factor

## Abstract

The insulin-like growth factor (IGF) pathway is thought to play an important role in brain development. Altered levels of IGFs and their signaling regulators have been shown in autism spectrum disorder (ASD) patients. In this study, we investigated whether coding region single-nucleotide polymorphisms (cSNPs) of the insulin receptor substrates (*IRS1* and *IRS2*), key mediators of the IGF pathway, were associated with ASD in Korean males. Two cSNPs (rs1801123 of *IRS1*, and rs4773092 of *IRS2*) were genotyped using direct sequencing in 180 male ASD patients and 147 male control subjects. A significant association between rs1801123 of *IRS1* and ASD was shown in additive (*p* = 0.022, odds ratio (OR) = 0.66, 95% confidence interval (CI) = 0.46–0.95) and dominant models (*p* = 0.013, OR = 0.57, 95% CI = 0.37–0.89). Allele frequency analysis also showed an association between rs1801123 and ASD (*p* = 0.022, OR = 0.66, 95% CI = 0.46–0.94). These results suggest that *IRS1* may contribute to the susceptibility of ASD in Korean males.

## 1. Introduction

Autism spectrum disorder (ASD) is a neurodevelopmental disorder characterized by deficits in social communication, impaired reciprocal social interaction, and repetitive patterns of behaviors or interests [1]. ASD has been reported to occur in approximately six out of 1000 births, affecting males and females in a ratio of 4:1 [2]. ASD has been found throughout the world across all racial, ethnic and social backgrounds. Although the cause of ASD remains elusive, accumulating evidence suggests that genetic factors play a prominent role in this disease. Heritability is estimated to be above 90% [3]. Inherited copy number variations (CNVs) and chromosomal abnormalities have shown to contribute to genetic vulnerability to ASD [4,5]. In addition, recent multiple genome-wide association studies suggest several common single-nucleotide polymorphisms (SNPs) as markers of ASD [6,7,8].

Previous studies have revealed the importance of the insulin-like growth factor (IGF) pathway in the development and maintenance of the central nervous system (CNS). During both development and adulthood, increased IGF1 and IGF2 levels were associated with increased neuronal complexity and impaired learning, suggested that IGFs support the activity-dependent neuronal plasticity underlying cognitive processes [9,10]. Moreover, recent studies have highlighted a significant role for IGF1 in complex social interactions [11,12]. It has been reported that levels of IGF1, IGF2, and IGF binding protein 3 (IGFBP3) were significantly increased in patients with ASD [13]. Furthermore, both cross-sectional and longitudinal studies have reported that a subset of ASD patients show age-dependent brain overgrowth [14,15]. Brain overgrowth at an early age could be caused by conditional overexpression of IGF1 in the brain, which is responsible for significant increases in brain volume during the embryonic and early postnatal period [16]. Indeed, the significant correlation between head circumference and IGF1 levels was also shown in ASD patients, but not in the controls [13]. On the other hand, other studies reported lower IGF1 levels in the cerebrospinal fluid (CSF) of autistic patients [17,18]. In addition, it was proposed that a reduced peripartum level of IGF1 due to genetic, epigenetic, or environmental factors may be a sentinel biomarker of increased probability of the later development of autism [19].

Although little is known about the biological function of the IGF pathway molecules in ASD, given the previous reports, we speculated that IGFs and their signaling regulator genes might be candidate genes involved in ASD. However, to our knowledge, there have not been any studies on the possible genetic association of IGFs or IGF signaling regulator genes with ASD. In this study, we focused on key molecules of the IGF pathway, insulin receptor substrate 1 (*IRS1*) and *IRS2*, which are the major cytosolic substrates of the IGF receptors and mediators for the downstream pathway processes [20,21].

Insulin has been regarded as primarily a metabolic signal, while IGFs has been implicated as an important mitogen and cell differentiation factor [22,23]. The IRS family contains several members (IRS1-6), of which IRS1 and IRS2 have been most widely studied. IRS1 and IRS2 regulate body weight control and glucose homeostasis [24]. They could also control body growth and peripheral insulin action. Thus, they have been suggested as markers of an active IGF pathway within tumors [25,26], although they are involved in insulin signaling. Indeed, polymorphisms of *IRS1* and/or *IRS2* have shown the significant associations with diabetes, glucose levels [27], and obesity [28], as well as with cancers, along with IGF signaling regulator genes [29,30,31]. Herein, we investigated the association of the coding region single-nucleotide polymorphisms (cSNP) of *IRS1* and *IRS2*, active markers of the IGF pathway, with ASD in Korean males.

## 2. Results

Two cSNPs of *IRS1* and *IRS2* were polymorphic, and the genotype distributions of the SNPs were in Hardy-Weinberg equilibrium (HWE) (*p* > 0.05; data not shown). We calculated the power of the sample size to verify our data using a genetic power calculator [32]. Considering a two-fold genotype relative risk, the sample powers of the SNPs were 0.900 (rs1801123, number of effective samples for 80% power = 148) and 0.967 (rs4773092, *n* = 110), respectively (α = 0.05). In addition, the sample powers of rs1801123 were 0.841 (*n* = 175) and 0.761 (*n* = 213) for a 1.9- and 1.8-fold relative risk, respectively. The sample powers of rs4773092 were 0.938 (*n* = 128) for a 1.9-fold relative risk, 0.889 (*n* = 154) for a 1.8-fold relative risk, and 0.889 (*n* = 189) for a 1.7-fold relative risk. Therefore, the results of our study had a significant power and sample size to detect the genotype relative risks up to 1.9-fold on rs1801123 and 1.8-fold on rs4773092.

As shown in Table 1, rs1801123 of *IRS1* was associated with ASD in additive (AG vs. GG vs. AA, *p* = 0.022, odds ratio (OR) = 0.66, 95% confidence interval (CI) = 0.46–0.95) and dominant models (*p* = 0.013, OR = 0.57, 95% CI = 0.37–0.89). The frequency of the genotypes containing the G allele (AG/GG, 36.7%) was decreased in the ASD patients compared to the control subjects (50.3%). In allele frequency analysis, we also found that rs1801123 was associated with ASD (*p* = 0.022, OR = 0.66, 95% CI = 0.46–0.94). The frequency of the G allele was lower in ASD patients (21.1%) than in control subjects (28.9%). This significance remained after the Bonferroni correction.

Interestingly, when we analyzed the differences between patients with autistic disorder and healthy individuals, rs1801123 of IRS1 showed a statistically more significant association (Table 2). The association was revealed in the additive (*p* = 0.0037, OR = 0.56, 95% CI = 0.37–0.83) and dominant models (*p* = 0.0041, OR = 0.50, 95% CI = 0.31–0.81). Allele frequency analysis also revealed a stronger association between rs1801123 and autistic disorder (*p* = 0.004, OR = 0.56, 95% CI = 0.38–0.84). The frequencies of the AG/GG genotypes (33.6% and 50.3% in patients with autistic disorder and control subjects, respectively) and the G allele (18.6% and 28.9%) were more remarkably decreased in patients with autistic disorder compared to control subjects.

## 3. Discussion

In our study, we found that rs1801123 of *IRS1* was significantly associated with ASD in Korean males. The G allele of rs1801123 contributed to a decreased risk of ASD and, particularly, the contribution was potently shown in patients with autistic disorder.

The IGF pathway plays an important role in regulating cell proliferation, differentiation and apoptosis, and, thus, IGFs and their signaling regulators have been studied in growth-, weight gain- , and obesity-related diseases [20,28,33,34,35]. IRS1 and IRS2 are key mediators of the IGF pathway [20,21]. Binding of IGFs to IGF receptors phosphorylates IRSs and triggers downstream cascades such as MAPK and PI3K/AKT signaling, which finally leads to cell proliferation and differentiation [20,21]. Thus, IRS1 and IRS2 together with IGFs and IGFRs have been involved in obesity, birth weight, diabetes mellitus, insulin sensitivity and cancer, showing the genetic associations of their polymorphisms [28,29,30,31,33,34].

In ASD patients, increased head growth, and particularly brain overgrowth in early life, has been reported with or without higher weights and body mass indexes (BMIs) Thus, several studies have suggested the involvement of growth-related hormones such as IGFs and their regulators, which lead to increased head growth and higher weights and BMIs, in the pathophysiology of autistic disorder/ASD [13,17,18]. Indeed, Mills et al. [13] reported increased levels of IGF1, IGF2, IGFBP3 and GHBP in the plasma of autistic disorder/ASD patients, and also showed a positive correlation between IGF1 level and head circumference in autistic disorder/ASD patients.

On the other hands, IGFs and their regulators have been also reported to play a role in growth retardation. Indeed, transgenic mice lacking *IRS1* showed prenatal and postnatal growth retardation [36,37]. In mice lacking *IRS2*, growth retardation was also observed, although it was minimal compared to mice lacking *IRS1* [36]. Moreover, in the brain, IGFs and their regulators are essential factors for normal brain growth and development, as well as synaptogenesis and myelination [16,19,38]. They directly affect the rate that oligodendrocytes promote myelination, and thus factors which relatively reduce the production or availability of IGFs could retard normal nerve programming [19]. Indeed, in early laboratory embryos, the addition of IGF-receptor inhibitors blocked the normal formation of midbrain neurons [39]. *IGF1* knockout mice had defective neurologic development [40]. Moreover, other studies on the relationship between IGF1 level and autistic patients reported lower IGF1 levels in the cerebrospinal fluid (CSF) of autistic patients, although the IGF1 levels of patients were compared to abnormal controls instead of normal controls due to ethical reasons that do not allow researchers to obtain CSF from normal control subjects [17,18]. Thus, relatively low activities of IGF pathway molecules such as IGF1, IRS1 and IRS2 may play a role as risk factors in the pathophysiology of autistic disorder/ASD, leading to the growth and development retardation. Hence, it is controversial which one among excessive activations and reduced availabilities of IGF-related factors is involved in the pathology of autistic disorder/ASD.

In the present study, we found that rs1801123 of *IRS1* was associated with ASD, and the association was shown more strongly in patients with autistic disorder. In particular, our results showed that the frequency of the minor G allele of rs1801123 was decreased in patients with autistic disorder/ASD; thus, the G allele of rs1801123 may contribute to a decreased risk of autistic disorder/ASD as a protective factor. In a previous study, carriers of the minor allele of rs1801123 (TG/GG) were reported to be associated with higher fasting plasma glucose and insulin levels [27]. Furthermore, the G allele of rs1801123 was associated with an increased risk of breast cancer in women carrying the *BRCA1* mutation [30], although in a recent study of the same group using a large set of *BRCA1* and *BRCA2* mutation carriers, its lack of association was revealed [41]. Moreover, the G allele of rs1801123 was significantly associated with lymph node involvement in estrogen-receptor-positive primary invasive breast cancer patients, who were treated with surgery and tamoxifen [29]. These reports indicated that the minor allele of rs1801123 may be involved in the increased product and ability of the IGFs, along with the activation of the insulin-related signaling pathway. Therefore, the decreased frequency of the minor allele of rs1801123 in autistic disorder/ASD patients in our study may contribute to relatively low activation of the IGF pathway molecules. Taken together, we postulated that the activation of IGF pathway molecules may be reduced in autistic disorder/ASD patients, although it is controversial as mentioned above. Also, the G allele of rs1801123 may play a role as a protective factor against the decreased activity of the IGF pathway in autistic disorder/ASD. Further studies are needed to determine how rs1801123 and the IGF pathway affect the pathophysiology of autistic disorder/ASD.

Our study is the first pilot to report an association of the *IRS1* with autistic disorder/ASD. The limitation of our study is that only one SNP of each *IRS1* and *IRS2* was selected and analyzed. Replication studies are needed to determine the association between the *IRS1* and autistic disorder/ASD, as well as the lack of association of *IRS2*, analyzing more polymorphisms in addition to rs1801123 and rs4773092. Moreover, as shown in our sample power analysis, our results have statistical confidence, only assuming a genotype relative risk up to 1.9-fold on rs1801123. Thus, the relatively small sample size limits the generalizability of the findings from the present study. Our findings are preliminary and need to be validated in further studies with larger sample sizes. Our work provides evidence that the *IRS1* gene may play a role in the pathophysiology of ASD.

## 4. Experimental Section

### 4.1. Subjects

One hundred eighty male ASD patients (mean age ± standard deviation (SD), 15.5 ± 4.8 years) and 147 healthy male individuals (39.9 ± 5.8 years) were enrolled in this study. ASD patients were diagnosed with ASDs by well-trained psychiatrists, child and adolescent specialists according to Diagnostic and Statistical Manual of Mental Disorders, 4th ed (DSM-IV) criteria [42], using available historical information from interviews and clinical records. Each ASD patient was also evaluated using the Childhood Autism Rating Scale (CARS) [43], one of the most widely used instrument to evaluate the developmental degree of autism, applying cut-off score of 30. The average of CARS score was 38.6 ± 5.8 (mean ± SD). The ASD group consisted of 137 patients with autistic disorder, 11 patients with Asperger’s disorder, and 32 patients with Pervasive Developmental Disorder-Not Otherwise Specified (PDD-NOS). A summary of clinical characteristics of ASD patients is provided in Table 3. The healthy adult controls were recruited from subjects who visited the hospital for routine health checkups. Controls were investigated to determine whether they or their first-degree relatives had psychiatric disturbances or previous psychiatric treatment through personal interviews. Only unaffected subjects with no psychiatric disorder or family history were included in this study.

All the ASD patients and control subjects were of Korean background. The present study was conducted in accordance with the guidelines of the Helsinki Declaration and was approved by the Ethics Review Committee of Medical Research Institute, Kyung Hee University Medical Center on 15 September 2004 (2004-09-15). Written informed consents were obtained from the parents or guardians of ASD patients and control subjects.

### 4.2. Single-Nucleotide Polymorphism (SNP) Selection and Genotyping

Of SNPs in the *IRS1* and *IRS2*, cSNPs were targeted and selected from the National Center for Biotechnology Information SNP database [44]. Some cSNPs alter a functionally important amino acid residue, and these are of interest for their potential links with phenotype. Other cSNPs may prove useful for their potential links to functional cSNPs via linkage disequilibrium mapping [45,46]. We selected common SNPs with a minor allele frequency of >0.1 in Chinese and Japanese populations, excluding SNPs without data on genotype frequency. Finally, we selected two cSNP (rs1801123 (Ala804Ala) of *IRS1*, and rs4773092 (Cys816Cys) of *IRS2*).

Genomic DNA was extracted from the whole blood of each subject using the High Pure PCR Template Preparation kit (Roche, Mannheim, Germany) following the manufacturer’s protocol. SNP genotyping was conducted with direct sequencing using the following primers for each SNP: rs1801123 in *IRS1* (sense, 5′-TCCTACTACTCATTGCCAAGATC-3′; antisense, 5′-CTATTGGTCTGAGCAGCTGTGT-3′), and rs4773092 in *IRS2* (sense, 5′-ATGTGGTGCGGTTCCAAGCTGT-3′; antisense, 5′-GCCAAAGTCGATGTTGATGTACT-3′). The PCR products were sequenced using the ABI PRISM 3730XL analyzer (PE Applied Biosystems, Foster City, CA, USA), and sequence data were then analyzed using SeqManII software (DNASTAR Inc., Madison, WI, USA).

### 4.3. Statistical Analysis

SNPStats [47] and SPSS 18.0 software (SPSS Inc., Chicago, IL, USA) were used to analyze the genetic data and the HWE. The association between SNP genotypes and ASD were estimated by computing the ORs and their 95% CIs with logistic regression analyses. In the logistic regression analysis for each SNP, the following models were used: codominant inheritance (that is, where the relative hazard differed between subjects with one minor allele and those with two minor alleles), dominant inheritance (subjects with one or two minor alleles had the same relative hazard for the disease), or recessive inheritance (subjects with two minor alleles were at increased risk of the disease). The chi-square test was used to compare allele frequencies between groups. To avoid chance findings due to multiple testing, a Bonferroni correction was applied by lowering the significance levels to *p* = 0.025 (*p* = 0.05/2) for two SNPs.

## Figures and Tables

**Table 1:** Multiple logistic regression analysis of *IRS1* and *IRS2* polymorphisms in autism spectrum disorder (ASD) patients and control subjects.

SNP	Model/Allele	Genotype	Control	ASD	OR (95% CI)	*p*
			*n* (%)	*n* (%)		
rs1801123	Additive	AA	73 (49.7)	114 (63.3)	1	
Ala804Ala		AG	63 (42.9)	56 (31.1)		
*IRS1*		GG	11 (7.5)	10 (5.6)	0.66 (0.46–0.95)	**0.022**
	Dominant	AA	73 (49.7)	114 (63.3)	1	
		AG/GG	74 (50.3)	66 (36.7)	0.57 (0.37–0.89)	**0.013**
	Recessive	AA/AG	136 (92.5)	170 (94.4)	1	
		GG	11 (7.5)	10 (5.6)	0.73 (0.30–1.76)	0.48
	Allele	A	209 (71.1)	284 (78.9)	1	
		G	85 (28.9)	76 (21.1)	0.66 (0.46–0.94)	**0.022**
rs4773092	Additive	AA	41 (27.9)	51 (28.3)	1	
Cys816Cys		AG	76 (51.7)	95 (52.8)		
*IRS2*		GG	30 (20.4)	34 (18.9)	0.96 (0.70–1.32)	0.8
	Dominant	AA	41 (27.9)	51 (28.3)	1	
		AG/GG	106 (72.1)	129 (71.7)	0.98 (0.60–1.59)	0.93
	Recessive	AA/AG	117 (79.6)	146 (81.1)	1	
		GG	30 (20.4)	34 (18.9)	0.91 (0.53–1.57)	0.73
	Allele	A	158 (53.7)	197 (54.7)	1	
		G	136 (46.3)	163 (45.3)	0.96 (0.70–1.31)	0.8

Bold characters represent statistically significant values (*p* < 0.025). ASD, autism spectrum disorder. 1—It is a statistical reference in our genetic analysis.

**Table 2:** Multiple logistic regression analysis of *IRS1* and *IRS2* polymorphisms in patients with autistic disorder and control subjects.

SNP	Model/allele	Genotype	Control	Autistic Disorder	OR (95% CI)	*p*
			*n* (%)	*n* (%)		
rs1801123	Additive	AA	73 (49.7)	91 (66.4)	1	
Ala804Ala		AG	63 (42.9)	41 (29.9)		
*IRS1*		GG	11 (7.5)	5 (3.6)	0.56 (0.37–0.83)	**0.0037**
	Dominant	AA	73 (49.7)	91 (66.4)	1	
		AG/GG	74 (50.3)	46 (33.6)	0.50 (0.31–0.81)	**0.0041**
	Recessive	AA/AG	136 (92.5)	132 (96.3)	1	
		GG	11 (7.5)	5 (3.6)	0.47 (0.16–1.38)	0.16
	Allele	A	209 (71.1)	223 (81.4)	1	
		G	85 (28.9)	51 (18.6)	0.56 (0.38–0.84)	**0.004**
rs4773092	Additive	AA	41 (27.9)	36 (26.3)	1	
Cys816Cys		AG	76 (51.7)	70 (51.1)		
*IRS2*		GG	30 (20.4)	31 (22.6)	1.08 (0.77–1.51)	0.64
	Dominant	AA	41 (27.9)	36 (26.3)	1	0.76
		AG/GG	106 (72.1)	101 (73.7)	1.09 (0.64–1.83)	
	Recessive	AA/AG	117 (79.6)	106 (77.4)	1	0.65
		GG	30 (20.4)	31 (22.6)	1.14 (0.65–2.01)	
	Allele	A	158 (53.7)	142 (51.8)	1	
		G	136 (46.3)	132 (48.2)	0.96 (0.70–1.31)	0.8

Bold characters represent statistically significant values (*p* < 0.025). 1—It is a statistical reference in our genetic analysis.

**Table 3:** Clinical characteristics of ASD patients and control subjects.

Characteristics	ASDs	Control
Total no. of subject	180	147
Age (mean ± SD, years)	15.5 ± 4.8	39.9 ± 5.8
CARS score	38.6 ± 5.8	
Autistic disorder (*n* = 137)	41.1 ± 4.3	
Asperger‘s disorder (*n* = 11)	30.7 ± 0.4	
PDD-NOS (*n* = 32)	30.9 ± 1.0	

ASD, autism spectrum disorder; CARS, Childhood Autism Rating Scale; PDD-NOS, Pervasive Developmental Disorder-Not Otherwise Specified.
